# Increasing the Vaccine Potential of Live *M*. *bovis* BCG by Coadministration with Plasmid DNA Encoding a Tuberculosis Prototype Antigen

**DOI:** 10.3390/vaccines2010181

**Published:** 2014-03-05

**Authors:** Nicolas Bruffaerts, Marta Romano, Olivier Denis, Fabienne Jurion, Kris Huygen

**Affiliations:** Scientific Institute of Public Health, Communicable and Infectious Diseases, Immunology, Brussels 1180, Belgium; E-Mails: nicolas.bruffaerts@wiv-isp.be (N.B.); marta.romano@wiv-isp.be (M.R.); olivier.denis@wiv-isp.be (O.D.); fabienne.jurion@wiv-isp.be (F.J.)

**Keywords:** tuberculosis, DNA vaccine, BCG, PPE44

## Abstract

The attenuated live *M*. *bovis* Bacille-Calmette-Guérin (BCG) is still the sole vaccine used against tuberculosis, but confers only variable efficacy against adult pulmonary tuberculosis (TB). Though no clear explanation for this limited efficacy has been given, different hypotheses have been advanced, such as the waning of memory T-cell responses, a reduced antigenic repertoire and the inability to induce effective CD8^+^ T-cell responses, which are known to be essential for latent tuberculosis control. In this study, a new BCG-based vaccination protocol was studied, in which BCG was formulated in combination with a plasmid DNA vaccine. As BCG is routinely administered to neonates, we have evaluated a more realistic approach of a simultaneous intradermal coadministration of BCG with pDNA encoding the prototype antigen, PPE44. Strongly increased T- and B-cell responses were observed with this protocol in C57BL/6 mice when compared to the administration of only BCG or in combination with an empty pDNA vector, as measured by Th1-type spleen cell cytokine secretion, specific IgG antibodies, as well as specific IFN-γ producing/cytolytic-CD8^+^ T-cells. Moreover, we observed a bystander activation induced by the coding plasmid, resulting in increased immune responses against other non-plasmid encoded, but BCG-expressed, antigens. In all, these results provide a proof of concept for a new TB vaccine, based on a BCG-plasmid DNA combination.

## 1. Introduction

Live *M*. *bovis* Bacille-Calmette-Guérin (BCG) is currently one of the most widely used vaccines (annually, 120 million vaccine doses worldwide, with four billion vaccinated to date), and still, the only available vaccine against tuberculosis (TB). Indeed, BCG has been administered to neonates in the context of the Expanded Program on Immunization (EPI) since 1974, as it confers protection against miliary TB and TB meningitis in young children with a reduced risk of disease development of 50% [1]. Moreover, its extensive safety record in humans, heat stability and low production cost makes it particularly attractive. BCG presents, however, a highly variable and insufficient protection efficacy against pulmonary TB, the most common and contagious form of the disease [2]. In 2012, 8.6 million new TB cases and 1.3 million TB deaths (among 0.3 million HIV-associated TB deaths) were estimated [3]. A clear explanation for the poor protective efficacy of BCG against pulmonary TB is still not available, though a number of studies have addressed different hypotheses, such as the waning of the memory T-cell response [4], the variability of the administered BCG strains [5], the responses to a more limited antigenic repertoire as compared to the one of *Mycobacterium tuberculosis* (*Mtb*) [6] and the influence of pre-existing immunity to antigens shared with non-tuberculous mycobacteria [7]. Another possible explanation for this poor efficacy is linked to the limited ability of the BCG vaccine to induce effective CD8^+^ T-cell responses compared to *Mtb*, probably because its lack virulent RD-1 antigens CFP-10 and ESAT-6, which are known to facilitate mycobacteria translocation to the cytosol [8]. Even if their precise contribution in host defense against *Mtb* remains unclear, the role of CD8^+^ T-cell responses in controlling *Mtb* growth, especially during latency, is considered essential [9]. CD8^+^ T-cells exert an antimycobacterial function by producing cytolytic and microbicidal effector molecules and also contribute to the activation of infected macrophages through their production of the Th1-type cytokines, IFN-γ and TNF-α [10,11].

In the quest for an efficient vaccine against TB, most strategies rely on the improvement of BCG by replacing it with other recombinant strains of attenuated mycobacteria or on prime-boost immunization protocols. The latter are based on attempts to enhance/boost previously BCG-induced immunity with subunit vaccines based on immunodominant antigens, either as viral-vectors, such as AdAg85A and MVA85A, or as recombinant fusion proteins from *Mtb* formulated in adjuvants promoting Th1-type responses [12].

Plasmid DNA-based vaccines are another class of promising sub-unit vaccines that can be used in the context of novel TB vaccines to generate MHC Class I and II-restricted immune responses [13]. When combined in a classical BCG-prime DNA-boost vaccination strategy, numerous preclinical studies have shown an increase of BCG potency against *Mtb* [14,15,16,17]. However, in most of these reports, protective efficacy was only measured during a short-term post-infection period. Alternatively, other studies showed increased specific CD4^+^ and CD8^+^ T-cell responses by priming with DNA and boosting with BCG [18,19,20]. Moreover, we have previously shown in a murine long-term survival study that priming with an Ag85A-encoding plasmid DNA prior to BCG vaccination could significantly increase BCG-induced protective efficacy, while boosting with the same plasmid did not [21].

Because of the wide clinical use of BCG in neonates, prior administration with a different vaccine is considered as an unrealistic goal. To our knowledge, there are no studies that attempted to directly mix a DNA vaccine with the live *M*. *bovis* BCG, instead of the classical prime-boost regimens. In the context of other diseases, some studies took advantage of the adjuvant properties of BCG, formulating DNA vaccines with BCG cell wall polysaccharide and/or nucleic acid fractions [22,23]. In these studies, enhanced cellular and humoral responses were induced, with the activation of TLR signaling pathways and Th1-type cytokine secretion. However, for optimal protective responses against *Mtb*, the viability of BCG is critical [24]. In this context, recent studies showed how live, but not killed, bacteria can induce significant expression of Type I IFNs and IL-1β from macrophages and dendritic cells, probably through bacterial mRNA sensing [25].

Here, we have evaluated the vaccine potential of intradermal co-administration of live BCG with a prototype tuberculosis DNA vaccine encoding PPE44 (Rv2770c), a putative virulence factor of *Mtb*. By differential mRNA display, it was found that this protein is overexpressed in virulent *Mtb* H37Rv, as compared to the attenuated *Mtb* H37Ra strain, and weakly expressed by BCG [26]. Furthermore, *ppe44* expression shows high quantitative variations in clinical isolates selected to represent the major phylogenetic lineages of the *M*. *tuberculosis* complex, and more specifically, strains of the Beijing type demonstrate high *ppe44* expression. PPE44-specific immune responses can be detected in mice acutely, chronically and latently infected with *Mtb*; the antigen contains well-characterized MHC Class II- and Class I-restricted epitopes and confers protection against *Mtb* H37Rv when administered as a protein or pDNA vaccine [27].

## 2. Experimental

### 2.1. Animals

Female C57BL/6 mice aged 6–8 weeks were bred and kept at the WIV-ISP experimental animal facilities (Ukkel site, Brussels), complying with the Belgian legislation that transposes European Directive 2009/41/EC, repealing Directive 90/219/EC (EC, 2009).

### 2.2. Vaccination Protocol

**Figure 1:**
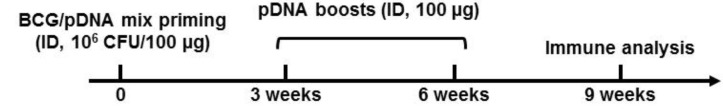
BCG/pDNA co-vaccination protocol. Priming was performed in adult mice with a mix of 10^6^ CFU live *M*. *bovis* BCG and 100 µg coding or non-coding plasmid DNA by the intradermal route in the tail (ID). After 3 weeks of resting, two further boosts at 3-week intervals were administered with 100 μg of plasmid at the same site. Two additional control groups consisted of one prime-immunized intradermally only with BCG (10^6^ CFU/mouse) and another intramuscularly (IM) immunized three times only with 100 µg coding plasmid at 3-week intervals. Animals were sacrificed 3 weeks after the second DNA boost to analyze cellular immune responses in spleen and inguinal lymph nodes and humoral responses in serum.

A schematic timeline representing the experimental protocol is depicted in Figure 1. Priming was performed in adult mice by the intradermal route (ID) with a fresh mix of 10^6^ CFU of live *M*. *bovis* BCG (strain Pasteur GL2) and 100 µg of non-coding plasmid DNA (namely, pV1J.ns-tPA [28]), or plasmid DNA encoding PPE44 (namely, pV1J.ns-tPA-PPE44) or plasmid DNA encoding OVA (namely, pCI-OVA, a generous gift of Dr. Joseph Thalhamer and Dr. Richard Weiss [29]). Intradermal injections were carried out 1–2 cm distal from the tail base with a maximum total volume of 100 µL. After 3 weeks of resting, two further intradermal boosts at 3-week intervals were administered with the same 100 μg plasmid at the same site. Two additional control groups consisted of mice primed intradermally with only BCG (10^6^ CFU/mouse) and of mice immunized intramuscularly (IM) three times with 100 µg coding plasmid at 3-week intervals. Intramuscular injections were performed in both quadriceps muscles with 2 × 50 μL, after anesthesia with ketamine/xylazine.

### 2.3. Cytokine Production

Throughout this study, the immunogenicity of BCG/pDNA co-vaccination was evaluated by *ex vivo* restimulation of splenocytes from vaccinated mice, sacrificed 3 weeks after the last immunization. Organs were removed aseptically and homogenized. Leucocytes (4 × 10^6^ cells/mL) were cultivated at 37 °C in a humidified CO_2_ incubator in round-bottomed microwell plates in a volume of 200 μL RPMI-1640 medium (Life Technologies, Carlsbad, CA, USA) supplemented with 5 × 10^−5^ M 2-mercaptoethanol, 1% penicillin-streptomycin and 10% fetal calf serum (FCS). Six mice per group were individually analyzed for spleen cell cytokine productions specific to purified recombinant *E*. *coli*-expressed PPE44 (Rv2770c), Ag85A (Rv3804c) and PstS-3 (Rv0928) purified in our unit [30], culture filtrate from *M*. *bovis* BCG and Imject^®^ Ovalbumin (Thermo Scientific, Waltham, MA, USA). The final concentration of the recombinant proteins and culture filtrate was 5 µg/mL. Furthermore, synthetic peptides at a final concentration of 10 µg/mL were used, namely a peptide spanning the predicted PPE44 D^b^ restricted epitope (amino acids 257–265) and the PPE44 I-A^b^ restricted epitope (amino acids 1–20) [27]. Spleen cell culture supernatants were harvested after 24 h (for IL-2) and 72 h (for IFN-γ, TNF-α and IL-17A), when peak values of the respective cytokines can be measured. Supernatants were stored frozen at −20 °C until analysis. IFN-γ was detected using an enzyme-linked immunosorbent assay (ELISA) with purified rat anti-mouse IFN-γ as the capture antibody and biotin-labelled rat anti-mouse IFN-γ as the detection antibody (BD Pharmingen, Franklin Lakes, NJ, USA). Plates were revealed with O-phenylenediamine dihydrochloride substrate (OPD; Sigma-Aldrich, St. Louis, MO, USA); the reaction was stopped with 1 M H_2_SO_4_, and the optical density was read at 490 nm. IL-2, TNF-α and IL-17A were detected using commercial ELISA kits (eBioscience, San Diego, CA, USA).

### 2.4. *In Vivo* CTL Activity Assessment by Adoptive Transfer of CFSE-Labelled Target Cells

One week after the second DNA boost, mice were intravenously injected with carboxyfluorescein diacetate (CFSE)-labelled target cells. Target cells were prepared as described before [31]. Briefly, spleens from naive C57BL/6 mice were removed aseptically and homogenized, washed in RPMI, resuspended at 20 × 10^6^ cells/mL in RPMI-10% FCS and incubated for 1 h at 37 °C/5% CO_2_, either alone or with 10 μg/mL of the D^b^-restricted peptide, PPE44_257–265_. After incubation, cells were washed, resuspended in RPMI at 20 × 10^6^ cells/mL and labelled for 10 min at 37 °C in the dark with succinimidyl ester of carboxyfluorescein diacetate (CFSE) (Sigma-Aldrich) either at 2 μM (unpulsed cells; CFSE^low^) or 20 μM (peptide-pulsed cells; CFSE^high^). The staining reaction was stopped by the addition of an equal volume of RPMI supplemented with 10% of FCS; cells were then washed and resuspended in PBS at 100 × 10^6^ cells/mL. To a 1:1 mix of CFSE^low^/CFSE^high^, 20 × 10^6^ cells were adoptively transferred. Adoptively transferred mice were sacrificed 18 h later, spleens removed and homogenized and the erythrocytes depleted by lysis in ammonium chloride solution, and the cells were washed and resuspended in PBS for acquisition on a FACSCalibur cytofluorometer. To evaluate the percentage of specific lysis, the ratio of CFSE^high^/CFSE^low^ in vaccinated mice was compared to the ratio in transferred naive control mice. For each experimental group, six mice were tested.

### 2.5. IgG Antibody Secretion

Sera of vaccinated mice were collected at the time of immune analysis. Levels of specific antibodies in individual sera were determined by ELISA. For that purpose, 96-well plates were coated overnight with the specific recombinant protein at 500 ng/well in borate buffer. Adsorption sites were saturated during 1 h with 5% skimmed milk in PBS. Serial two-fold dilutions of sera were added for 2 h. Then, a peroxidase-labelled secondary rat anti-murine IgG (LO-MK-1 for IgG, purchased at Experimental Immunology Unit, Université Catholique de Louvain, Brussels, Belgium) was added for 1.5 h. Plates were developed following the same protocol used for cytokine detection, as previously described.

### 2.6. Statistical Analysis

GraphPad Prism software was used to perform statistical analysis. One-way ANOVA and Tukey’s post-test were performed to demonstrate statistical differences.

## 3. Results and Discussion

### 3.1. BCG/PPE44-Encoding pDNA Co-Vaccination Enhances Specific Th1-Type Cytokine Secretion

**Figure 2:**
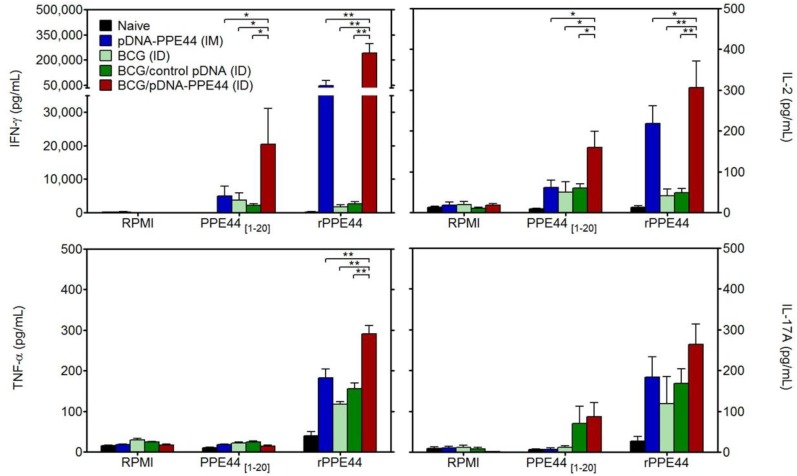
Specific Th1-type cytokine production induced by BCG/pDNA co-vaccination. IFN-γ, IL-2, TNF-α and IL-17A levels (pg/mL) were measured by ELISA in 24 h (IL-2) or 72 h (IFN-γ, TNF-α and IL-17A) culture supernatants of splenocytes from vaccinated C57BL/6 mice, after restimulation with the PPE44 MHC Class II-restricted synthetic peptide spanning amino acids 1–20 (PPE44_[1–20]_) or the recombinant PPE44 protein (rPPE44). RPMI, non-stimulated cells. ID, intradermally. IM, intramuscularly. Data are presented as the mean ± standard error of the mean and representative of two independent experiments, including six mice per group. * *p <* 0.05; ** *p <* 0.005.

The production of IFN-γ, IL-2, TNF-α and IL-17A was evaluated in the culture supernatants of spleen cells isolated from vaccinated and control groups after restimulation with recombinant PPE44 or the PPE44_1–20_ peptide. We have previously described that PPE44_1–20_ is an immunodominant T CD4^+^ epitope recognized after BCG immunization, as well as after DNA vaccination [27]. As shown in Figure 2, PPE44-specific IFN-γ and IL-2 levels measured in the spleen of BCG/pDNA-PPE44 (ID) co-vaccinated C57BL/6 mice were greatly increased and significantly higher than the levels measured in mice of the control groups immunized with BCG alone or with a combination of BCG with a non-coding, empty pDNA vector. The levels induced by the BCG/pDNA-PPE44 combination were also significantly higher than the levels induced after three intramuscular pDNA-PPE44 administrations. TNF-α could be detected in cultures stimulated with recombinant PPE44 protein, but not with PPE44_1–20_ peptide. In addition, increased TNF-α levels were observed in BCG/pDNA-PPE44 immunized mice compared to the levels achieved after vaccination with BCG, BCG combined to non-coding pDNA or intramuscular vaccination with pDNA-PPE44. Finally, no significant differences in specific IL-17A levels were observed between the different experimental groups.

### 3.2. BCG/PPE44-Encoding pDNA Co-Vaccination Enhances Effective Cytolytic and IFN-γ-Producing CD8^+^ T-Cell Responses

**Figure 3:**
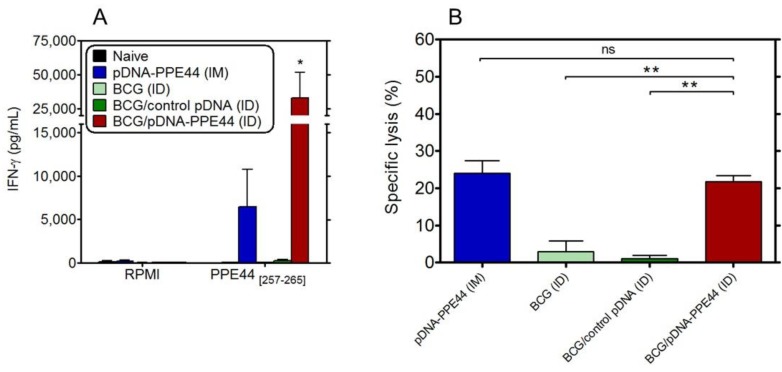
Specific CD8^+^ T-cell responses induced by BCG/pDNA co-vaccination. (**A**) IFN-γ levels (pg/mL) were measured by ELISA in 72 h culture supernatant of splenocytes cultured with the PPE44 MHC Class I-restricted short peptide spanning amino acids 257–265 (PPE44_[257–265]_). RPMI, non-stimulated cells. Data are presented as the mean ± standard error of the mean and representative of two independent experiments, including six mice per group. * *p <* 0.05. (**B**) *In vivo*-specific cytotoxic T lymphocyte (CTL) activity. Unpulsed splenocytes (carboxyfluorescein diacetate (CFSE)^l^^ow^) and peptide-pulsed splenocytes (CFSE^high^) from naive mice were intravenously transferred to the immunized mice 18 h before flow cytometry analysis. Splenocytes were pulsed with the PPE44 MHC Class I-restrictedshort peptide spanning amino acids 257–265. The percentages of specific lysis in the immunized mice were compared to the ratio in transferred naive control mice. Data represent the mean ± standard error of the mean (*n =* 3–6/group). ** *p <* 0.005. ID, intradermally. IM, intramuscularly.

Specific IFN-γ was detected in spleen cell culture supernatants from mice vaccinated intramuscularly with pDNA-PPE44 and mice co-vaccinated with BCG/pDNA-PPE44 after stimulation with the synthetic PPE44_257–265_ peptide (Figure 3A). We have previously reported that PPE44_257–265_ spans a predicted D^b^-restricted epitope and on the *in vivo* cytotoxic T lymphocyte (CTL) activity against PPE44_257–265_ in C57BL/6 mice vaccinated with pDNA encoding PPE44 [27]. Hence, we verified whether BCG/pDNA-PPE44 co-vaccination could also induce effective CD8^+^ T-cells by assessing their *in vivo* cytolytic activity. PPE44_257–265_ peptide-pulsed, CFSE-labelled spleen cells from naive C57BL/6 mice were adoptively transferred as targets in vaccinated mice. As expected, a specific lysis of about 23% was measured by flow cytometry in mice vaccinated three times intramuscularly with pDNA-PPE44. In addition, a similar level of specific lysis was observed in mice intradermally coimmunized with BCG/pDNA-PPE44 (Figure 3B). In contrast, no PPE44-specific CTL activity could be detected in mice vaccinated with BCG alone or in mice vaccinated with BCG/empty vector, confirming the inability of the attenuated *M*. *bovis* BCG vaccine to induce detectable CD8^+^ T-cell responses.

### 3.3. BCG/PPE44-Encoding pDNA Co-Vaccination Enhances Mycobacteria-Specific Responses against Non-Plasmid-Encoded but BCG-Expressed Antigens

**Figure 4:**
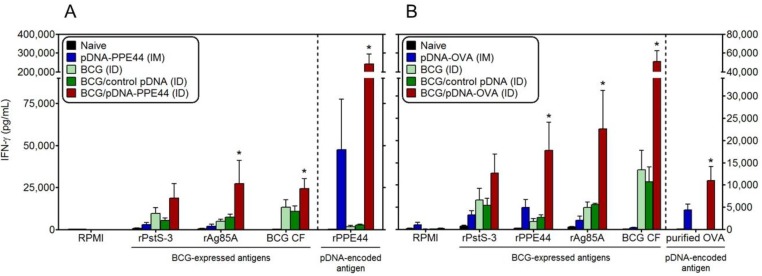
Mycobacteria-specific IFN-γ responses against non plasmid-encoded, but BCG-expressed, antigens after BCG/pDNA co-vaccination. IFN-γ (pg/mL) was measured by ELISA in 72 h culture supernatant of spleen cells cultured with mycobacterial recombinant proteins PstS-3 and Ag85A, BCG culture filtrate (BCG CF) and with recombinant PPE44 (**A**) or purified ovalbumin (**B**). Data are presented as the mean ± standard error of the mean and representative of two independent experiments, including six mice per group. * *p <* 0.05 in comparison with BCG (ID) and BCG/control pDNA (ID) groups.

In order to find out whether the BCG/pDNA-PPE44 combination could affect the responses specific to antigens expressed by BCG other than PPE44, spleen cells were stimulated with two other BCG antigens, recombinant PstS-3 (Rv0928) and recombinant Ag85A (Rv3804c), or BCG culture filtrate. PstS-3 is one of the three putative phosphate transport receptors of *Mtb*, known to be an immunodominant B- and T-cell antigen of *M*. *bovis* BCG in H-2^b^ haplotype mice [32]. PstS-3 is a potential tuberculosis vaccine candidate, and we have previously shown that mice vaccinated with PstS-3 DNA demonstrated significant and sustained reduction in bacterial CFU numbers in spleen and lungs for three months after *Mtb* challenge [33]. As shown in Figure 4A, the combination of BCG with pDNA-PPE44 increased the IFN-γ response to recombinant PstS-3 antigen about two-fold. IFN-γ responses to another immunodominant BCG antigen with a very promising vaccine potential, the mycolyl-transferase Ag85A, were even more strongly increased by the BCG/pDNA-PPE44 combination, and responses against BCG culture filtrate (in which Ag85A is one of the most abundant proteins) were equally augmented. Mice co-vaccinated with BCG and control DNA showed the same response as mice vaccinated with BCG alone.

### 3.4. Bystander Activation of IFN-γ Responses against BCG-Expressed Antigens by BCG/OVA-Encoding pDNA Co-Vaccination

In order to find out more about the bystander activation observed in the BCG/pDNA-PPE44 group, mice were vaccinated with a combination of BCG and a plasmid DNA encoding an unrelated, non-mycobacterial antigen, *i.e.*, ovalbumin (OVA). As shown in Figure 4B, BCG co-vaccination with pDNA-OVA also induced increased IFN-γ responses specifically to the three BCG-encoded antigens, PstS-3, PPE44 and Ag85A, and to BCG culture filtrate. IFN-γ levels were two-fold higher against recombinant PstS-3, eight-fold higher against PPE44 and about four-fold higher against Ag85A in mice that had been vaccinated with the BCG/pDNA-OVA combo, as compared to mice only vaccinated with BCG or BCG/control pDNA. As expected, IFN-γ responses to purified OVA were only detected in mice vaccinated with pDNA-OVA or with the BCG/pDNA-OVA combination, but not in naive or BCG vaccinated mice. IFN-γ levels in the supernatant of unstimulated spleen cells were highest in mice vaccinated three times with pDNA-OVA (in blue), which may have contributed to some extent to the mycobacteria-specific IFN-γ responses to PstS-3, PPE44 and Ag85A observed in this group. Interestingly, OVA-specific IFN-γ titers in the BCG/pDNA-OVA animals were higher than in the mice vaccinated only with pDNA-OVA, although both groups had received the same plasmid dose three times.

### 3.5. BCG/PPE44-Encoding pDNA or BCG/pDNA-OVA Co-Vaccination also Enhances Antigen-Specific Antibody Responses

**Figure 5:**
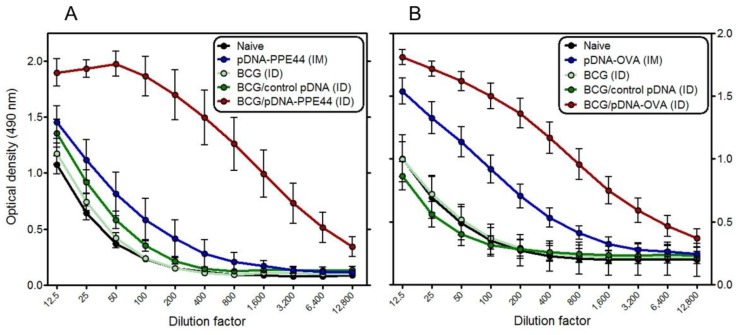
Specific antibody production induced by BCG/pDNA co-vaccination. Anti-PPE44 (**A**) and anti-OVA (**B**) IgG-isotype antibodies were measured by ELISA in serum harvested three weeks after the last immunization. Optical density levels of serial two-fold serum dilutions are presented as the mean ± standard error of the mean and representative of two independent experiments, including five to six mice per group.

Total anti-PPE44 and anti-OVA IgG levels were measured in sera (Figure 5). BCG/coding-pDNA coimmunization increased the specific antibody levels to the corresponding antigen as compared to the levels observed in both BCG control groups and to levels observed after intramuscular vaccination with coding pDNA. Anti-PPE44 end-point titers in BCG/pDNA-PPE44 coimmunized mice were about thirty-fold higher than in BCG or BCG/control pDNA vaccinated mice and about nine-fold higher than in pDNA-PPE44 vaccinated mice. Anti-OVA end-point titers in coimmunized mice were about twenty-fold and nine-fold higher when compared, respectively, to BCG or BCG/control pDNA vaccinated mice and to coding pDNA vaccinated mice. Anti-PPE44 and anti-OVA end-point titers measured in BCG vaccinated mice and BCG/control pDNA co-vaccinated mice were not significantly different from the titers measured in naive mice. As for the IFN-γ responses, antibody titers in the BCG/pDNA coimmunized animals were higher than in the mice vaccinated with coding plasmid.

## 4. Conclusions

BCG improvement strategies are needed and under analysis for the development of a more efficient tuberculosis vaccine. Th1-type responses are essential in anti-tuberculosis protection, but CD8^+^ T-cell responses also play a very important role, particularly in the protection against reactivation of a latent tuberculosis infection [9,34,35]. In this study, we have shown that the weak potential of BCG to trigger MHC Class I-restricted immune responses can be overcome by coimmunization with a plasmid DNA vaccine. Induction of strong CD8^+^ T-cell responses is perhaps the most important hallmark of DNA vaccines, making them particularly attractive as vaccines against viral and other intracellular pathogens [12].

We have described a new BCG/pDNA co-vaccination protocol that is effective in inducing significantly higher CD4^+^ Th1 and IFN-γ producing/cytolytic CD8^+^ T-cell responses in C57BL/6 mice as compared to animals immunized with BCG alone or in a combination with a non-coding pDNA vector. As a prototype antigen, we used the PPE44 (Rv2770c) antigen, a putative virulence factor of *Mtb*, against which specific immune responses can be detected in mice acutely, chronically and latently infected [27]. The PPE protein family of *Mtb*, characterized by Pro-Pro-Glu motifs near the *N*-terminus of their sequence, includes 69 proteins rich in glycine and together with the PE (Pro-Glu) protein family, represents approximately 10% of the coding genes scattered throughout the *Mtb* genome. Proteins of this family have been found to be T-cell immunodominant antigens in animal models and humans, eliciting very high cellular responses and making them of potential interest for vaccine development, e.g., PPE18 (Rv1196), found in the fusion polyprotein of the TB subunit vaccine candidate, Mtb72F [36,37], and PPE14 (Rv0915c) [38]. PPE18 and PPE14 share 14 identical amino acids in their NH_2_-terminal 20-amino acid sequence with PPE44, a region which encodes the I-A^b^ restricted Th1 epitope, against which strongly increased responses were found in the BCG/pDNA-PPE44 combination. It is possible that cross-reactive responses against PPE14 and PPE18 are induced by the BCG/pDNA-PPE44 immunization (which could increase the protective efficacy), but more work is needed to formally demonstrate this.

An antigen dose effect could be partially responsible for the increased PPE44-specific responses, as this protein is relatively weakly expressed in BCG [39]. Antigen load contributes to the development of specific CD8^+^ T-cell responses, in terms of kinetics and magnitude [40]. Furthermore, the differentiation of long-lived memory CD8^+^ T-cells *in vivo* was reported to correlate with high antigen dose and the stability of T-cell-DC contacts [41]. In this context, it is worth mentioning that a two hundred-fold higher dose of *M*. *bovis* BCG than of *Mtb* is needed to induce similar CD8^+^ T-cell responses [8]. On the other hand, highly increased immune responses can also be detected in mice and pigs immunized with a combination of BCG and pDNA encoding the mycolyl-transferase Ag85A, which is among the most strongly expressed proteins in the culture filtrate of the BCG vaccine [42].

A strong bystander activation was observed with the BCG/pDNA-PPE44 protocol, resulting in higher responses also to other BCG-expressed antigens. Furthermore, this bystander activation was observed in mice vaccinated with the BCG/pDNA-OVA combination, but not in mice vaccinated with the BCG/control pDNA, suggesting that antigen-specific cytokine responses were elicited *in vivo* by the booster vaccinations with coding pDNAs, which created an immunostimulatory Th1 cytokine environment that amplified responses induced by the BCG priming to these antigens. Bystander activation of T-cells has been best described for CD8^+^ T-cells, and in this compartment, the release of IFN and IFN inducers leads to the production of IL-15, which mediates the proliferation of CD8^+^ T-cells [43,44]. Activated CD4^+^ T-cells of unrelated specificity can also undergo bystander activation, probably involving IL-2 and IL-7 as potential cytokine mediators [45]. It is tantalizing to speculate that similar bystander activation could also be stimulated in other combination protocols using plasmid DNA boosters and live, attenuated bacterial or viral vaccines.

Besides the bystander activation caused by the plasmid DNA boosters, BCG also exerted adjuvant effects on the plasmid DNA-induced responses. Thus, OVA-specific IFN-γ titers were higher in BCG/pDNA-OVA than in pDNA-OVA vaccinated mice, although both groups had received the same plasmid dose three times. Differences in administration route, *i.e*., intradermal *versus* intramuscular, are unlikely to play a role, as we have found comparable immune responses to the pDNA administered by either route [46]. The BCG cell wall is composed of several pathogen associated molecular patterns (PAMPs) that are able to interact with different pathogen recognition receptors (PRRs), such as TLR2, TLR4 and TLR9, resulting in the induction of innate immune responses [47]. Plasmid DNA vaccines also have intrinsic adjuvant properties, because they can activate TLR9 through their bacterial CpG motifs and TBK1-dependent innate immune signaling pathways through their double-stranded structure [48]. Therefore, it is tempting to speculate that there might be synergies on TLR-induced innate immune responses provided by BCG and pDNA, knowing, for example, that TLR9 regulates Th1 responses and cooperates with TLR2 in mediating optimal resistance to *Mtb* [49]. Moreover, TLR9 signaling seems to be critical for the induction of effective CD8^+^ T-cell responses through cross-priming following the initial pDNA immunization [50,51]. On the other hand, CD4^+^ Th1 responses and T CD8^+^ responses were not different in animals vaccinated with BCG alone or in combination with non-coding pDNA, indicating that the adjuvant effects of the vector backbone were minimal [28].

BCG is routinely administered by the intradermal route, and we have confirmed here that this route can also be used for plasmid DNA vaccines [52]. This intradermal route is particularly attractive, as skin-associated lymphoid tissues contain a wide variety of specialized cells able to enhance immune responses, such as dermal (langerin expressing) dendritic cells and macrophages, keratinocytes and Langerhans cells [53]. 

In conclusion, this study has given a proof of principle that the routine BCG immunization protocol could be improved by formulating BCG with pDNA vaccines encoding protective *Mtb* antigens. The prototype antigen encoded by the pDNA vaccine used in this study could be replaced by other antigens or combination of antigens, such as the latency-associated antigens, against which only poor responses are induced by BCG vaccination [6]. Another approach could be to potentiate CD8^+^ T-cell responses by using a plasmid DNA vaccine encoding a fusion of immunodominant MHC Class I-restricted epitopes. Although Th1 type immune responses are considered to be essential, the precise correlates of protection against TB are still not fully defined, and excepting studies in non-human primates, there is no proper animal model that can reproduce the reactivation of latent tuberculosis. Long-term survival experiments in mice may give some information, and these experiments are actually in progress.

## References

[1] Trunz B.B., Fine P., Dye C. (2006). Effect of BCG vaccination on childhood tuberculous meningitis and miliary tuberculosis worldwide: A meta-analysis and assessment of cost-effectiveness. Lancet.

[2] Fine P.E. (1995). Variation in protection by BCG: Implications of and for heterologous immunity. Lancet.

[3] World Health Organization (2013). Global Tuberculosis Report.

[4] Weir R.E., Gorak-Stolinska P., Floyd S., Lalor M.K., Stenson S., Branson K., Blitz R., Ben-Smith A., Fine P.E., Dockrell H.M. (2008). Persistence of the immune response induced by BCG vaccination. BMC Infect. Dis..

[5] Ritz N., Hanekom W.A., Robins-Browne R., Britton W.J., Curtis N. (2008). Influence of BCG vaccine strain on the immune response and protection against tuberculosis. FEMS Microbiol. Rev..

[6] Lin M.Y., Geluk A., Smith S.G., Stewart A.L., Friggen A.H., Franken K.L., Verduyn M.J., van Meijgaarden K.E., Voskuil M.I., Dockrell H.M. (2007). Lack of immune responses to *Mycobacterium tuberculosis* DosR regulon proteins following *Mycobacterium bovis* BCG vaccination. Infect. Immun..

[7] Weir R.E., Black G.F., Nazareth B., Floyd S., Stenson S., Stanley C., Branson K., Sichali L., Chaguluka S.D., Donovan L. (2006). The influence of previous exposure to environmental mycobacteria on the interferon-gamma response to bacille Calmette-Guerin vaccination in southern England and northern Malawi. Clin. Exp. Immunol..

[8] Ryan A.A., Nambiar J.K., Wozniak T.M., Roediger B., Shklovskaya E., Britton W.J., Fazekas de St Groth B., Triccas J.A. (2009). Antigen load governs the differential priming of CD8 T cells in response to the bacille Calmette Guerin vaccine or *Mycobacterium tuberculosis* infection. J. Immunol..

[9] Van Pinxteren L.A., Cassidy J.P., Smedegaard B.H., Agger E.M., Andersen P. (2000). Control of latent *Mycobacterium tuberculosis* infection is dependent on CD8 T cells. Eur. J. Immunol..

[10] Tascon R.E., Stavropoulos E., Lukacs K.V., Colston M.J. (1998). Protection against *Mycobacterium tuberculosis* infection by CD8^+^ T cells requires the production of gamma interferon. Infect. Immun..

[11] Bruns H., Meinken C., Schauenberg P., Harter G., Kern P., Modlin R.L., Antoni C., Stenger S. (2009). Anti-TNF immunotherapy reduces CD8^+^ T cell-mediated antimicrobial activity against *Mycobacterium tuberculosis* in humans. J. Clin. Investig..

[12] Romano M., Huygen K. (2012). An update on vaccines for tuberculosis-there is more to it than just waning of BCG efficacy with time. Exp. Opin. Biol. Ther..

[13] Liu M.A. (2011). DNA vaccines: An historical perspective and view to the future. Immunol. Rev..

[14] Derrick S.C., Yang A.L., Morris S.L. (2004). A polyvalent DNA vaccine expressing an ESAT6-Ag85B fusion protein protects mice against a primary infection with *Mycobacterium tuberculosis* and boosts BCG-induced protective immunity. Vaccine.

[15] Lu J., Wang C., Zhou Z., Zhang Y., Cao T., Shi C., Chen Z., Chen L., Cai C., Fan X. (2011). Immunogenicity and protective efficacy against murine tuberculosis of a prime-boost regimen with BCG and a DNA vaccine expressing ESAT-6 and Ag85A fusion protein. Clin. Dev. Immunol..

[16] Cervantes-Villagrana A.R., Hernandez-Pando R., Biragyn A., Castaneda-Delgado J., Bodogai M., Martinez-Fierro M., Sada E., Trujillo V., Enciso-Moreno A., Rivas-Santiago B. (2013). Prime-boost BCG vaccination with DNA vaccines based in beta-defensin-2 and mycobacterial antigens ESAT6 or Ag85B improve protection in a tuberculosis experimental model. Vaccine.

[17] Tan K., Liang J., Teng X., Wang X., Zhang J., Yuan X., Fan X. (2013). Comparison of BCG prime-DNA booster and rBCG regimens for protection against tuberculosis. Hum. Vaccin Immunother..

[18] Feng C.G., Palendira U., Demangel C., Spratt J.M., Malin A.S., Britton W.J. (2001). Priming by DNA immunization augments protective efficacy of *Mycobacterium bovis* Bacille Calmette-Guerin against tuberculosis. Infect. Immun..

[19] Ferraz J.C., Stavropoulos E., Yang M., Coade S., Espitia C., Lowrie D.B., Colston M.J., Tascon R.E. (2004). A heterologous DNA priming-Mycobacterium bovis BCG boosting immunization strategy using mycobacterial Hsp70, Hsp65, and Apa antigens improves protection against tuberculosis in mice. Infect. Immun..

[20] Dou J., Wang Y., Yu F., Yang H., Wang J., He X., Xu W., Chen J., Hu K. (2012). Protection against *Mycobacterium tuberculosis* challenge in mice by DNA vaccine Ag85A-ESAT-6-IL-21 priming and BCG boosting. Int. J. Immunogenet..

[21] Romano M., D’Souza S., Adnet P.Y., Laali R., Jurion F., Palfliet K., Huygen K. (2006). Priming but not boosting with plasmid DNA encoding mycolyl-transferase Ag85A from *Mycobacterium tuberculosis* increases the survival time of *Mycobacterium bovis* BCG vaccinated mice against low dose intravenous challenge with *M. tuberculosis* H37Rv. Vaccine.

[22] Zhang S., Guo Y.J., Sun S.H., Wang K.Y., Wang K.H., Zhang Y., Zhu W.J., Chen Z.H., Jiang L. (2005). DNA vaccination using Bacillus Calmette-Guerin-DNA as an adjuvant to enhance immune response to three kinds of swine diseases. Scand. J. Immunol..

[23] Sun J., Hou J., Li D., Liu Y., Hu N., Hao Y., Fu J., Hu Y., Shao Y. (2013). Enhancement of HIV-1 DNA vaccine immunogenicity by BCG-PSN, a novel adjuvant. Vaccine.

[24] Orme I.M. (1988). Induction of nonspecific acquired resistance and delayed-type hypersensitivity, but not specific acquired resistance in mice inoculated with killed mycobacterial vaccines. Infect. Immun..

[25] Sander L.E., Davis M.J., Boekschoten M.V., Amsen D., Dascher C.C., Ryffel B., Swanson J.A., Muller M., Blander J.M. (2011). Detection of prokaryotic mRNA signifies microbial viability and promotes immunity. Nature.

[26] Rindi L., Peroni I., Lari N., Bonanni D., Tortoli E., Garzelli C. (2007). Variation of the expression of *Mycobacterium tuberculosis* ppe44 gene among clinical isolates. FEMS Immunol. Med. Microbiol..

[27] Romano M., Rindi L., Korf H., Bonanni D., Adnet P.Y., Jurion F., Garzelli C., Huygen K. (2008). Immunogenicity and protective efficacy of tuberculosis subunit vaccines expressing PPE44 (Rv2770c). Vaccine.

[28] Huygen K., Content J., Denis O., Montgomery D.L., Yawman A.M., Deck R.R., DeWitt C.M., Orme I.M., Baldwin S., D’Souza C. (1996). Immunogenicity and protective efficacy of a tuberculosis DNA vaccine. Nat. Med..

[29] Stoecklinger A., Grieshuber I., Scheiblhofer S., Weiss R., Ritter U., Kissenpfennig A., Malissen B., Romani N., Koch F., Ferreira F. (2007). Epidermal langerhans cells are dispensable for humoral and cell-mediated immunity elicited by gene gun immunization. J. Immunol..

[30] Romano M., Roupie V., Wang X.M., Denis O., Jurion F., Adnet P.Y., Laali R., Huygen K. (2006). Immunogenicity and protective efficacy of tuberculosis DNA vaccines combining mycolyl-transferase Ag85A and phosphate transport receptor PstS-3. Immunology.

[31] Romano M., Denis O., D’Souza S., Wang X.M., Ottenhoff T.H., Brulet J.M., Huygen K. (2004). Induction of *in vivo* functional D^b^-restricted cytolytic T cell activity against a putative phosphate transport receptor of *Mycobacterium tuberculosis*. J. Immunol..

[32] Lefevre P., Braibant M., de Wit L., Kalai M., Roeper D., Grotzinger J., Delville J.P., Peirs P., Ooms J., Huygen K. (1997). Three different putative phosphate transport receptors are encoded by the *Mycobacterium tuberculosis* genome and are present at the surface of *Mycobacterium bovis* BCG. J. Bacteriol..

[33] Tanghe A., Lefevre P., Denis O., D’Souza S., Braibant M., Lozes E., Singh M., Montgomery D., Content J., Huygen K. (1999). Immunogenicity and protective efficacy of tuberculosis DNA vaccines encoding putative phosphate transport receptors. J. Immunol..

[34] Chen C.Y., Huang D., Wang R.C., Shen L., Zeng G., Yao S., Shen Y., Halliday L., Fortman J., McAllister M. (2009). A critical role for CD8 T cells in a nonhuman primate model of tuberculosis. PLoS Pathog..

[35] Villarreal-Ramos B., McAulay M., Chance V., Martin M., Morgan J., Howard C.J. (2003). Investigation of the role of CD8^+^ T cells in bovine tuberculosis *in vivo*. Infect. Immun..

[36] Von E.K., Morrison R., Braun M., Ofori-Anyinam O., De Kock E., Pavithran P., Koutsoukos M., Moris P., Cain D., Dubois M.C. (2009). The candidate tuberculosis vaccine Mtb72F/AS02A: Tolerability and immunogenicity in humans. Hum. Vaccin..

[37] Spertini F., Audran R., Lurati F., Ofori-Anyinam O., Zysset F., Vandepapeliere P., Moris P., Demoitie M.A., Mettens P., Vinals C. (2013). The candidate tuberculosis vaccine Mtb72F/AS02 in PPD positive adults: A randomized controlled phase I/II study. Tuberculosis.

[38] Skeiky Y.A., Alderson M.R., Ovendale P.J., Lobet Y., Dalemans W., Orme I.M., Reed S.G., Campos-Neto A. (2005). Protection of mice and guinea pigs against tuberculosis induced by immunization with a single *Mycobacterium tuberculosis* recombinant antigen, MTB41. Vaccine.

[39] Bonanni D., Rindi L., Lari N., Garzelli C. (2005). Immunogenicity of mycobacterial PPE44 (Rv2770c) in *Mycobacterium bovis* BCG-infected mice. J. Med. Microbiol..

[40] Russell M.S., Iskandar M., Mykytczuk O.L., Nash J.H., Krishnan L., Sad S. (2007). A reduced antigen load *in vivo*, rather than weak inflammation, causes a substantial delay in CD8^+^ T cell priming against *Mycobacterium bovis* (Bacillus Calmette-Guerin). J. Immunol..

[41] Henrickson S.E., Perro M., Loughhead S.M., Senman B., Stutte S., Quigley M., Alexe G., Iannacone M., Flynn M.P., Omid S. (2013). Antigen availability determines CD8^+^ T cell-dendritic cell interaction kinetics and memory fate decisions. Immunity.

[42] Bruffaerts N., Vandermeulen G., Romano M., Préat V., Stockhofe-Zurwieden N., Huygen K. (2014).

[43] Tough D.F., Borrow P., Sprent J. (1996). Induction of bystander T cell promiferation by viruses and type I interferon *in vivo*. Science.

[44] Zhang X., Sun S., Hwang I., Tough D.F., Sprent J. (1998). Potent and selective stimulation of memory-phenotype CD8^+^ T cells *in vivo* by IL-15. Immunity.

[45] Di Genova G., Savelyeva N., Suchaki A., Thirdborough S.M., Stevenson F.K. (2010). Bystander stimulation of activated CD4^+^ T cells of unrelated specificity following a booster vaccination with tetanus toxoid. Eur. J. Immunol..

[46] Bruffaerts N., Romano M., Denis O., Jurion F., Huygen K. (2014).

[47] Von Meyenn F., Schaefer M., Weighardt H., Bauer S., Kirschning C.J., Wagner H., Sparwasser T. (2006). Toll-like receptor 9 contributes to recognition of *Mycobacterium bovis* Bacillus Calmette-Guerin by Flt3-ligand generated dendritic cells. Immunobiology.

[48] Desmet C.J., Ishii K.J. (2012). Nucleic acid sensing at the interface between innate and adaptive immunity in vaccination. Nat. Rev. Immunol..

[49] Bafica A., Scanga C.A., Feng C.G., Leifer C., Cheever A., Sher A. (2005). TLR9 regulates Th1 responses and cooperates with TLR2 in mediating optimal resistance to *Mycobacterium tuberculosis*. J. Exp. Med..

[50] De Brito C., Tomkowiak M., Ghittoni R., Caux C., Leverrier Y., Marvel J. (2011). CpG promotes cross-presentation of dead cell-associated antigens by pre-CD8α^+^ dendritic cells. J. Immunol..

[51] Pavlenko M., Leder C., Moreno S., Levitsky V., Pisa P. (2007). Priming of CD8^+^ T-cell responses after DNA immunization is impaired in TLR9- and MyD88-deficient mice. Vaccine.

[52] Elnekave M., Furmanov K., Hovav A.H. (2011). Intradermal naked plasmid DNA immunization: Mechanisms of action. Exp. Rev. Vaccines.

[53] Heath W.R., Carbone F.R. (2013). The skin-resident and migratory immune system in steady state and memory: Innate lymphocytes, dendritic cells and T cells. Nat. Immunol..

